# Burden among family caregivers of children and adolescents followed for diabetes

**DOI:** 10.1192/j.eurpsy.2025.1598

**Published:** 2025-08-26

**Authors:** S. Kolsi, A. W. Noureddine, A. Bouaziz, A. Labyadh, K. Medhaffar, K. Zitoun, W. Abbes

**Affiliations:** 1 Psychiatry, University Hospital, Gabes, Tunisia

## Abstract

**Introduction:**

Children and adolescents are a fairly vulnerable population for diabetes.

Caregivers of children and adolescents with diabetes were most involved in their care.

Measuring the burden endured by caregivers is a good indicator of the negative repercussions on caregivers when caring for their loved ones.

**Objectives:**

- Determine the level of burden among family caregivers of children and adolescents with diabetes.
- Identify factors associated with a high level of burden.

**Methods:**

The study was conducted at the University Hospital of Gabès, in the pediatrics and internal medicine departments, as well as in outpatient clinics with caregivers of children and adolescents during the period from March 2024 to May 2024.

We collected sociodemographic and clinical data for each caregiver and child or adolescent followed for diabetes.

We used :

Burden scale (Zarit): explores the psychological, physical and social impact of patient care on the caregiver. The higher the score, the greater the burden. A score above 61 means a severe burden.

**Results:**

The study included 32 caregivers. The mean age was 35.55 years with extremes of 22 and 55 years and the sex ratio (M/F) was 0.6.

Workload was shared equally by 66% of caregivers (65.7%; n=21). The majority of the sample (62.5%) did not seem to have experienced family conflicts related to the caregiver’s role.

Among the participants, the majority, 78.1% (n=25), presented symptoms of psychological distress. A high level of stress was perceived by 43.7% of caregivers and satisfaction was noted by 34.4%.

A mean burden score was 35.59 (SD =15.37) with extremes from 8 to 59. We reported that 40.6% of caregivers presented a moderate to severe burden and 34.4% presented a light to moderate burden.

According to our results, heavy burden was statistically more common among caregivers with unequal workload sharing (p=0.006) and family conflict (p=0.01).

We found a statistically significant correlation between burden and symptoms of psychological distress (p=0.001), daily stress level (p=0.001) and overall satisfaction (p=0.001).

**Conclusions:**

Our study shows that the burden endured by caregivers of diabetic children and adolescents represents a real issue in the care of these patients.

Several factors seem to be inherent to the disease, the caregiver and the social context.

For this reason, it is imperative to develop specific support programs for family caregivers of diabetic children and adolescents. These programs should include interventions to reduce burden.

**Disclosure of Interest:**

None Declared

